# A three-parameter online nomogram for diabetic retinopathy risk in primary care: development and external validation in an independent cohort of type 2 diabetes

**DOI:** 10.3389/fendo.2026.1809663

**Published:** 2026-04-15

**Authors:** Jian Yang, Zhifu Zhang, Yaping Zhang, Bingsong Xie, Xuelan Li, Hairong Zhou

**Affiliations:** 1 Department of General Medicine, Longhua District People’s Hospital, Shenzhen, China; 2 Shantou University Medical College, Shantou, China; 3 Baijiahu Community Health Service Center, Nanjing, China

**Keywords:** diabetic retinopathy, interactive nomogram, prediction model, primary care, risk stratification

## Abstract

**Background:**

Diabetic retinopathy (DR) remains a leading cause of blindness among working-age adults, yet scalable risk stratification tools tailored to primary care are lacking—particularly in underserved settings where specialized examinations are unavailable. We aimed to develop and externally validate a pragmatic, web-based nomogram for DR risk prediction using only routinely collected electronic health record (EHR) variables in community-dwelling individuals with type 2 diabetes (T2DM).

**Methods:**

This retrospective cohort study analyzed EHR data from two independent Chinese populations. The primary cohort comprised 1,215 T2DM patients from 45 community health centers in Shenzhen, randomly split into training (n=851) and internal validation (n=364) sets. An external validation cohort of 329 patients was obtained from a center in Nanjing. Candidate predictors were screened via univariate analysis and least absolute shrinkage and selection operator (LASSO) regression within the training set. Selected variables were entered into multivariable logistic regression to construct a nomogram, which was deployed as an interactive web application. Model performance was assessed using the area under the receiver operating characteristic curve (AUC-ROC), calibration plots, decision curve analysis (DCA), and clinical impact curves (CIC).

**Results:**

Three predictors—diabetes duration, HbA1c, and high body mass index (BMI ≥24 kg/m², Chinese standard)—were retained in the final model. The model demonstrated robust discrimination: AUC was 0.77 (95% CI: 0.73–0.81) in the training set, 0.79 (0.73–0.85) in internal validation, and 0.81 (0.75–0.87) in external validation. Calibration was adequate, with non-significant Hosmer–Lemeshow tests (P > 0.05) and Brier scores below 0.15 across all cohorts. DCA confirmed positive net benefit over a wide range of threshold probabilities (10–95%), and CIC revealed a 1:1 ratio between predicted and observed DR cases at risk thresholds above 40%.

**Conclusion:**

This three-parameter online nomogram provides a simple, readily implementable tool for DR risk stratification in primary care. Its robust external validation in an independent cohort and reliance on variables universally available in EHRs position it as a cost-effective solution to bridge the screening gap and enable timely specialist referral for high-risk T2DM patients.

## 1 Introduction

Diabetic retinopathy (DR)—one of the most severe microvascular complications of type 2 diabetes mellitus (T2DM)—accounts for 8–12% of global blindness cases (1). Individuals with diabetes have a 25−fold higher risk of vision loss than non−diabetics, making DR the leading cause of irreversible visual impairment in the working−age population worldwide (2–4). According to the International Diabetes Federation (IDF) 2022 Atlas, approximately 103.12 million adults had DR globally in 2021 (22.27% of 537 million diabetics), a figure projected to reach 160.5 million by 2045 (5).

The disease burden is particularly severe in China, where rural DR prevalence (34.0%) far exceeds that in urban areas (18.7%). Alarmingly, only 10% of rural DR patients receive timely treatment, and about 70% of all diabetic individuals never undergo ocular screening (6, 7). The insidious progression of DR often leads to under−appreciation of its harm: once proliferative DR (PDR) develops, treatment costs quadruple, and 26% of untreated patients progress to blindness within two years (8). Current DR screening relies on specialized techniques—fundus photography (9), optical coherence tomography (OCT) (10), and fluorescein angiography (11)—but the limited availability of these resource−intensive examinations in primary care settings underscores an urgent need for community−adapted risk stratification tools.

Although machine learning (ML)−based DR prediction models have achieved considerable progress, their clinical implementation faces three critical obstacles:(1)Data limitations – Most models are developed using tertiary hospital cohorts and lack validation in heterogeneous community populations (12, 13).(2)Variable inaccessibility – Existing models often depend on retinal imaging or complex biomarkers (e.g., plasma adipokines) that are unavailable in primary care (14–16).(3)Insufficient external validation – Over 80% of current DR prediction models lack multi−center external validation, raising concerns about generalizability (17, 18).

Furthermore, China-focused studies have predominantly concentrated on urban tertiary hospitals, thereby neglecting the distinct risk profiles of community populations—who constitute 62% of all diabetic patients—characterized by infrequent glucose monitoring, irregular laboratory testing, and referral delays (19).

To address these gaps, we developed and validated a web-based interactive nomogram for DR risk prediction using EHR data from 45 community health centers affiliated with the Shenzhen Longhua District People’s Hospital Group and the Nanjing Jiangning Baijiahu Community Health Service Center. This model is designed to assist primary care providers in efficiently identifying high-risk DR patients, thereby providing evidence-based support for precision prevention within tiered healthcare systems.

## 2 Materials and methods

### 2.1 Data sources

We conducted a retrospective cohort study using electronic health record (EHR) data from two independent populations.

Primary cohort – The baseline population consisted of 1,313 T2DM patients registered between 1 January and 31 December 2024 at 45 community health centers under the Shenzhen Longhua District People’s Hospital Group. All patients had complete baseline clinical metrics. DR status was determined by tracking fundus imaging screening records (primarily fundus photography) up to 30 June 2025. After excluding 98 patients with missing key baseline variables or lacking follow-up fundus results, 1,215 eligible subjects were included and randomly allocated (7:3) to the model training (n = 851) and internal validation (n = 364) sets.

External validation cohort – An independent EHR dataset of 329 T2DM patients from the Nanjing Jiangning Baijiahu Community Health Service Center (same period) served for external validation.

**Figure 1:**
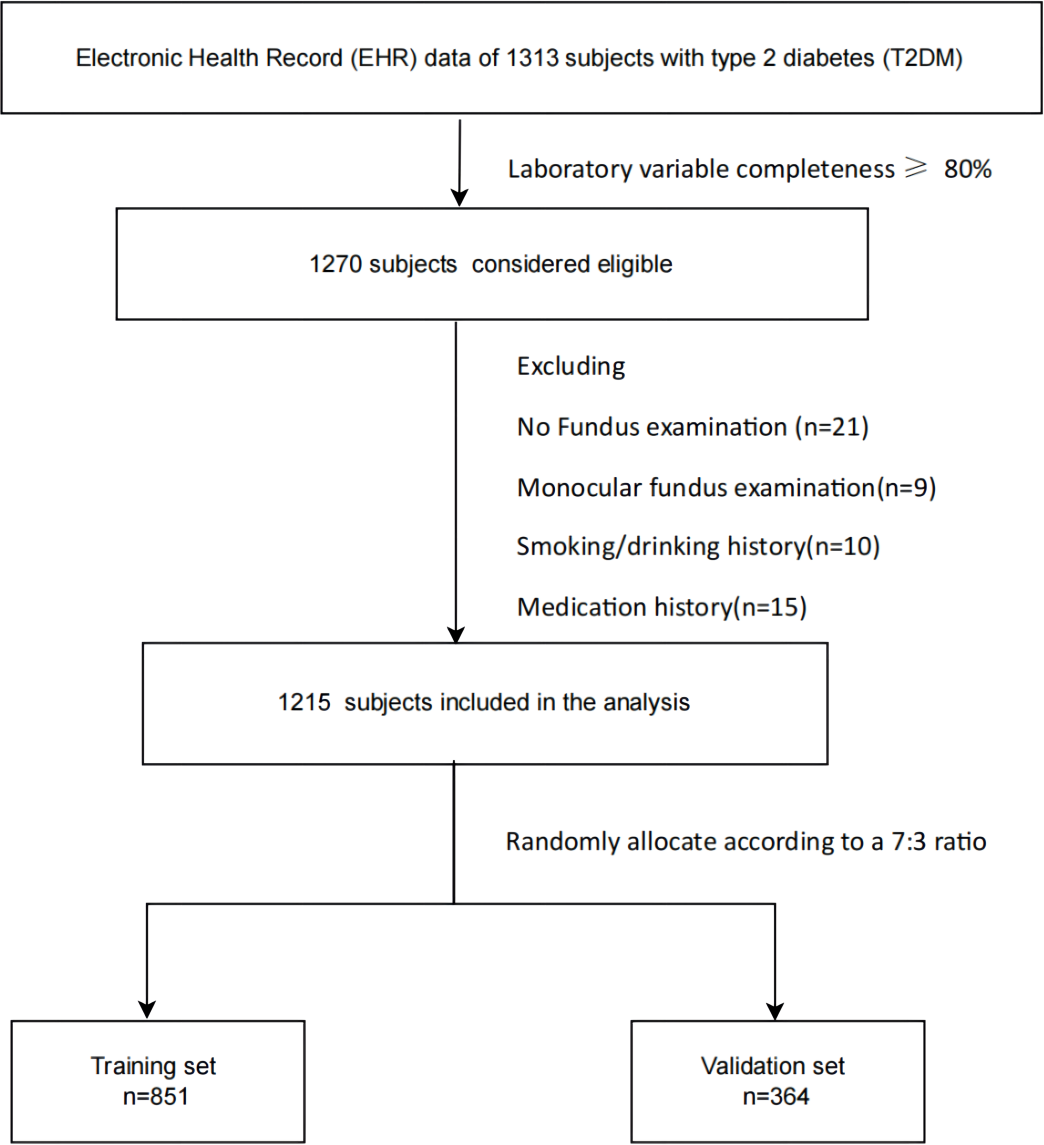
Flow diagram of participant selection and cohort assembly.

Participant selection is detailed in **Figure 1**. The study protocol was approved by the Shenzhen Longhua District People’s Hospital Ethics Committee (Approval No: LHHR-EC [2025]041). Formal data access authorization was obtained via a written agreement with the Data Governance Committee of the Nanjing Jiangning Baijiahu Community Health Service Center (Approval ID: BJH-SJZL-2025-016). All procedures adhered to the ethical principles of the Declaration of Helsinki and relevant international guidelines for retrospective data analysis. Individual informed consent was waived because de-identified retrospective data were used.

### 2.2 Diagnostic criteria

Type 2 diabetes mellitus (T2DM) was diagnosed according to the 2024 American Diabetes Association criteria. This was primarily confirmed by the presence of the corresponding ICD-10 code (E11) in the electronic health record (EHR). Additionally, medication records were reviewed to support the diagnosis (e.g., documented use of oral glucose-lowering agents, absence of insulin dependence within the first year of diagnosis, and no history of gestational diabetes).

Diabetic retinopathy (DR) was graded based on the internationally recognized Early Treatment Diabetic Retinopathy Study (ETDRS) scale (2018 revision) (20). The primary outcome was defined as the presence of any level of DR, including mild non-proliferative DR (NPDR), to maximize sensitivity for early detection in primary care settings. Assessment was performed using 7-field stereo fundus photography and independently evaluated by two senior retinal specialists. Inter-rater agreement between the two specialists was high (κ=0.92), indicating excellent reliability in DR grading.

Hypertension was defined per the 2018 ESC/ESH guidelines as repeated resting blood pressure measurements ≥140/90 mmHg or the use of documented antihypertensive treatment (21).

Cardiovascular disease (CVD) and stroke were confirmed based on the 2013 ACC/AHA criteria (22). Confirmation was established via a history of myocardial infarction, records of coronary revascularization procedures, or the presence of CT/MRI-confirmed ischemic stroke lesions.

Obesity was classified according to the 2025 Lancet criteria (23) into preclinical obesity, defined as a body mass index (BMI) ≥24 kg/m² (using the Chinese standard) without evidence of organ dysfunction, and clinical obesity, defined as a BMI ≥28 kg/m² or the presence of adiposity-related organ damage. For the purpose of this study, a BMI ≥24 kg/m² was uniformly termed ‘High BMI’ and used as the predictor variable in the risk model, whereas the clinical obesity definition (≥28 kg/m²) was retained only for diagnostic classification. We adopted this binary threshold (≥24 kg/m²) to align with the Chinese national guideline for overweight in Asian populations and to maximize clinical simplicity for point-of-care risk stratification in primary care settings.

### 2.3 Inclusion/exclusion criteria

Inclusion criteria were: (1) adult patients (≥18 years) with a confirmed diagnosis of T2DM; (2) availability of complete bilateral fundus screening results; and (3) ≥80% completeness for all clinical and laboratory variables of interest.

Exclusion criteria comprised: (1) non-T2DM diabetes types (ICD-10 codes E10 or O24, corroborated by clinical notes and medication history); (2) severe hepatic or renal dysfunction (eGFR <30 mL/min/1.73 m² or ALT/AST >3 times the upper limit of normal); (3) pregnancy or lactation; and (4) confounding ocular conditions (advanced glaucoma with visual field loss, cataract graded LOCS III ≥3, wet age-related macular degeneration, or prior vitrectomy/panretinal photocoagulation).

### 2.4 Data collection

The primary outcome was a binary classification of diabetic retinopathy (DR vs. non-DR). Variables extracted from the electronic health records (EHRs) encompassed a range of demographic, clinical, and laboratory parameters. These included basic demographic information (sex, age), diabetes-specific characteristics (diabetes duration), and lifestyle factors (smoking and alcohol history). We also collected data on comorbidities, including hypertension, cardiovascular disease (CVD), stroke, and High BMI, as well as the use of key medications such as angiotensin-converting enzyme inhibitors/angiotensin II receptor blockers (ACEI/ARB), statins, and sodium-glucose cotransporter-2 inhibitors (SGLT2i).

Laboratory parameters comprised the lipid profile (triglycerides [TG], total cholesterol [TC], high-density lipoprotein cholesterol [HDL-C], and low-density lipoprotein cholesterol [LDL-C]), hepatic and renal function markers (alanine aminotransferase [ALT], aspartate aminotransferase [AST], total bilirubin [TBIL], uric acid [UA], creatinine [Cr], blood urea nitrogen [BUN], and estimated glomerular filtration rate [eGFR]), and urinary markers (urinary microalbumin [UMA], urinary creatinine [UCr], and urinary albumin-to-creatinine ratio [UACR]). Glycemic control was assessed by fasting blood glucose (FBG) and glycated hemoglobin (HbA1c). Additionally, complete blood count (CBC) components and derived inflammatory ratios were extracted, including the neutrophil-to-lymphocyte ratio (NLR), platelet-to-lymphocyte ratio (PLR), lymphocyte-to-monocyte ratio (LMR), systemic immune-inflammation index (SII), and the triglyceride-glucose index (TyG).

### 2.5 Statistical analysis

All statistical analyses were performed using SPSS version 27.0 (IBM Corp., Armonk, NY, USA) and R software version 4.4.2 (R Foundation for Statistical Computing, Vienna, Austria). SPSS was primarily used for data management, descriptive statistics, and baseline comparisons. All predictive modeling, including feature selection, model building, validation, and visualization, was conducted in R. Specifically, the following R packages were employed: glmnet for LASSO regression, rms for nomogram construction and calibration plots, pROC for receiver operating characteristic (ROC) curve analysis, and rmda for decision curve analysis (DCA) and clinical impact curve (CIC) analysis.

Continuous variables were assessed for normality using the Shapiro-Wilk test. Normally distributed variables are presented as mean ± standard deviation and were compared using independent t-tests. Non-normally distributed variables are presented as median with interquartile range (IQR) and were compared using the Mann-Whitney U test. Categorical variables are presented as percentages and were compared using the chi-squared test.

Feature selection and model development were conducted using the following three-step process, with all steps strictly confined to the training set to prevent data leakage:

First, univariate logistic regression analysis was performed on the training set only to identify variables associated with diabetic retinopathy (DR) at a significance level of P < 0.05.

Subsequently, the significant variables from the univariate analysis were subjected to least absolute shrinkage and selection operator (LASSO) regression, again using only the training set. The optimal tuning parameter (λ) was determined via 10-fold cross-validation based on the minimum criteria. This step aimed to perform variable selection and regularization to prevent overfitting by shrinking the coefficients of less contributive variables to zero.

Finally, variables with non-zero coefficients from the LASSO regression were included in a multivariable logistic regression model to build the final prediction model. The results of the multivariable model are presented as odds ratios (ORs) with 95% confidence intervals (CIs).

The final model was presented as a nomogram. To enhance clinical usability, we developed an interactive, web-based version of this nomogram that allows for real-time risk calculation. The model’s performance was evaluated in terms of discrimination, calibration, and clinical utility. Discrimination, which refers to the model’s ability to distinguish between patients with and without DR, was assessed using the area under the receiver operating characteristic curve (AUC-ROC). Calibration, which measures the agreement between predicted probabilities and observed outcomes, was assessed using calibration plots and the Hosmer-Lemeshow test, with a non-significant P-value (P > 0.05) indicating good calibration. Clinical utility was evaluated using decision curve analysis (DCA), which quantifies the net benefit of using the model across different threshold probabilities, and clinical impact curves (CIC), which plot the number of true positives and false positives across thresholds. A two-tailed P-value < 0.05 was considered statistically significant for all analyses.

## 3 Results

### 3.1 Intergroup comparison of baseline characteristics

Baseline clinical and laboratory characteristics of participants in the training and internal validation sets are summarized in **Tables 1**, **2**. No statistically significant differences (P > 0.05) were observed between the two groups for demographic variables (sex, age, diabetes duration), comorbidities and lifestyle factors (DR prevalence, hypertension, CVD, stroke history, overweight/obesity, smoking/alcohol use), medication use (ACEI/ARB, statins, SGLT2i), or the majority of laboratory parameters—including complete blood count, hepatic/renal function, glycemic/lipid profiles (including HbA1c), inflammatory ratios (NLR, LMR, PLR, SII), and TyG index. The sole exception was platelet count (PLT), which showed a statistically significant but clinically negligible difference (P = 0.043).

**Table 1:** Demographic and clinical characteristics of the training and internal validation sets.

Variables	Training set (n=851)	Validation set (n=364)	P value
sex			0.891
Female	366 (43%)	155 (42.6%)	
Male	485 (57%)	209 (57.4%)	
Duration			0.27
<5 years	322 (37.8%)	148 (40.7%)	
5-10 years	227 (26.7%)	102 (28%)	
10-15 years	125 (14.7%)	45 (12.4%)	
15-20years	86 (10.1%)	35 (9.6%)	
≥20years	91 (10.7%)	34 (9.3%)	
DR (yes,n%)	205 (24.1%)	82 (22.5%)	0.557
Hypertension (yes,n%)	419 (49.2%)	182 (50%)	0.807
CVD (yes,n%)	87 (10.2%)	38 (10.4%)	0.909
Stroke (yes,n%)	127 (14.9%)	52 (14.3%)	0.774
High BMI (yes,n%)	408 (47.9%)	178 (48.9%)	0.76
Smoking (yes,n%)	280 (32.9%)	113 (31%)	0.526
Alcohol (yes,n%)	226 (26.6%)	96 (26.4%)	0.947
ACEI/ARB (yes,n%)	225 (26.4%)	102 (28%)	0.569
Statins (yes,n%)	250 (29.4%)	108 (29.7%)	0.918
SGLT2i (yes,n%)	297 (34.9%)	124 (34.1%)	0.78
Age (years,IQR)	58 (51,66)	57 (51,65)	0.442

DR, diabetic retinopathy; CVD, Cardiovascular disease; ACEI/ARB, Angiotensin-converting enzyme inhibitors/Angiotensin II receptor blockers; SGLT2i, Sodium-glucose cotransporter-2 inhibitors; IQR, interquartile range.

**Table 2:** Laboratory parameters of the training and internal validation sets.

Variables	Training set (n=851)	Validation set (n=364)	P value
FBG (mmol/l,IQR)	7.18 (6.18,8.4)	7.15 (6.18,8.26)	0.81
TBIL (μmol/L,IQR)	11.8 (9.4,15.4)	12 (9.1,15.1)	0.75
ALT (U/L,IQR)	21 (15,29)	20 (15,29)	0.755
AST (U/L,IQR)	20 (17,24)	20 (17,24.1)	0.782
TG (mmol/L,IQR)	1.45 (0.98,2.08)	1.395 (0.94, 2.09)	0.579
TC (mmol/L,IQR)	4.67 (3.95, 5.45)	4.69 (3.99, 5.49)	0.46
HDL-C (mmol/L,IQR)	1.21 (1.03, 1.41)	1.23 (1.06, 1.43)	0.292
LDL-C (mmol/L,IQR)	3.05 (2.36, 3.73)	3.085 (2.37, 3.69)	0.702
BUN (mmol/L,IQR)	5.73 (4.87, 6.81)	5.715 (4.77, 6.92)	0.968
Cr (μmol/L,IQR)	68.8 (57.5, 81.8)	67.7 (57.5, 81.18)	0.991
GFR (mL/min/1.73m²,IQR)	97 (83.18, 108.5)	98.5 (83.64, 110)	0.464
UA (μmol/L,IQR)	336 (280, 400)	338.5 (284, 410)	0.638
HbA1c (%,IQR)	6.9 (6.2,7.8)	6.9 (6.2,7.8)	0.666
UMA (mg/L,IQR)	19.3 (9.32, 51.3)	19.27 (8.89, 43.97)	0.609
UCr (umol/L,IQR)	10275 (7236, 14719)	10487.5 (7093, 14501)	0.952
UACR (mg/g,IQR)	14.98 (7.31, 41.02)	12.03 (6.06, 30.38)	0.214
WBC (×10^9^/L,IQR)	6.21 (5.09,7.27 )	6.13 (5.23,7.36 )	0.974
ANC (×10^9^/L,IQR)	3.66 (2.89,4.45 )	3.6 (2.84,4.42)	0.396
ALC (×10^9^/L,IQR)	1.91 (1.57,2.33)	1.97 (1.60,2.43)	0.23
AMC (×10^9^/L,IQR)	0.33 (0.27,0.41)	0.35 (0.27,0.43 )	0.089
RBC (×10¹²/L,IQR)	4.8 (4.48,5.15 )	4.83 (4.50,5.18)	0.483
HGB (g/L,IQR)	145 (134.00,155.00)	144 (135.00,154.75)	0.659
PLT (×10^9^/L,IQR)	218 (187.00,259.00 )	226.5 (192.00,266.75)	0.043
MPV (fL,IQR)	9.3 (8.70,10.00 )	9.3 (8.60,10.00)	0.171
NLR (IQR)	1.89 (1.46,2.41 )	1.78 (1.42,2.38)	0.132
LMR (IQR)	5.76 (4.51,7.39 )	5.76 (4.49,7.28 )	0.887
PLR (IQR)	114.29 (93.89,142.02)	117.51 (91.01,144.75)	0.642
SII (IQR)	406.5 (305.02,555.44)	405.71 (297.46,557.13)	0.58
TyG (IQR)	9.04 (8.59,9.48)	9.01 (8.50,9.44)	0.671

FBG, Fasting blood glucose;TBIL,Total bilirubin; ALT, Alanine aminotransferase; AST, Aspartate aminotransferase; TG,riglycerides;TC,Total cholesterol; HDL-C, High-density lipoprotein cholesterol; LDL-C, Low-density lipoprotein cholesterol; BUN, Blood urea nitrogen; Cr, Creatinine; eGFR, Estimated glomerular filtration rate; UA, Uric acid;HbA1c, Glycated hemoglobin;UMA, Urinary microalbumin; UCr, Urinary creatinine; UACR, Urinary albumin-to-creatinine ratio;WBC, White Blood Cell Count;ANC, Absolute Neutrophil Count;ALC, Absolute Lymphocyte Count;AMC, Absolute Monocyte Count;RBC, Red Blood Cell Count;HGB, Hemoglobin;PLT, Platelet Count;MPV, Mean Platelet Volume;NLR, Neutrophil-to-lymphocyte ratio; PLR, Platelet-to-lymphocyte ratio; LMR, Lymphocyte-to-monocyte ratio; SII, Systemic Immune-Inflammation; TyG, Triglyceride-glucose;IQR, interquartile range.

### 3.2 Screening of DR predictors in the training set

**Figure 2:**
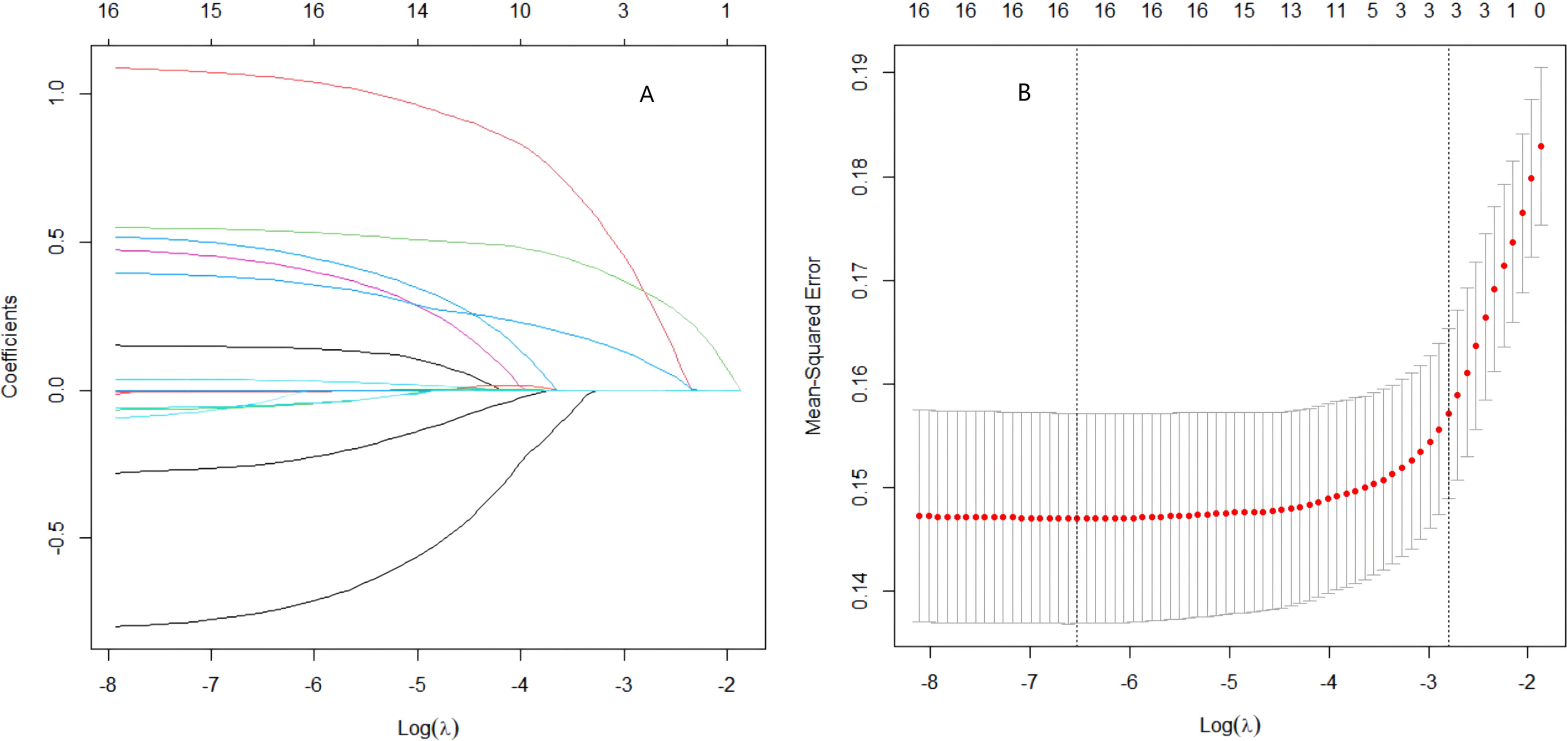
LASSO regression for feature selection. **(A)** Cross-validation deviance plot for λ selection. **(B)** Coefficient shrinkage paths of 17 candidate variables.

The feature selection process identified three key predictors for diabetic retinopathy: longer diabetes duration, elevated HbA1c, and high BMI. This final predictor set was derived from an initial pool of 17 variables that were significantly associated with DR in univariate analysis (P < 0.05; see **Table 3** for the full list). To refine the model and prevent overfitting, we applied LASSO regression with 10-fold cross-validation to these candidates (**Figures 2A, B**). The optimal regularization (λ = 0.06077) yielded non-zero coefficients only for the three aforementioned variables, all of which are well-established in DR pathogenesis.

**Table 3:** Univariate analysis of variables associated with diabetic retinopathy in the training set.

Variables	OR	Lower	Upper	P value
Sex	0.680	0.496	0.932	0.017
Age	1.017	1.002	1.032	0.029
Duration	1.838	1.629	2.075	0.000
Hypertension	1.082	0.790	1.482	0.623
CVD	1.771	1.104	2.843	0.018
Stroke	2.105	1.409	3.143	0.000
High BMI	3.038	2.177	4.239	0.000
Smoking	1.641	1.189	2.266	0.003
Alcohol	0.832	0.578	1.198	0.324
ACEI/ARB	1.169	0.823	1.658	0.383
Statins	1.342	0.959	1.879	0.086
SGLT2i	1.411	1.022	1.949	0.037
FBG	1.185	1.109	1.265	0.000
TBIL	0.983	0.951	1.015	0.292
ALT	0.994	0.982	1.005	0.278
AST	0.992	0.972	1.013	0.458
TG	0.990	0.899	1.090	0.834
TC	1.092	0.951	1.254	0.212
HDL-C	1.083	0.811	1.447	0.588
LDL-C	1.097	0.936	1.287	0.253
BUN	1.120	1.030	1.218	0.008
Cr	1.001	0.994	1.007	0.875
GFR	0.994	0.986	1.001	0.089
UA	1.000	0.998	1.001	0.791
HbA1c	1.462	1.315	1.625	0.000
UMA	1.001	1.000	1.001	0.011
UCr	1.000	1.000	1.000	0.294
UACR	1.000	1.000	1.000	0.293
WBC	1.030	0.946	1.122	0.491
ANC	1.120	0.998	1.257	0.054
ALC	0.754	0.580	0.981	0.036
AMC	0.902	0.260	3.126	0.871
RBC	0.827	0.616	1.112	0.208
HGB	0.992	0.982	1.002	0.114
PLT	1.002	0.999	1.004	0.211
MPV	0.908	0.779	1.057	0.212
NLR	1.231	1.056	1.435	0.008
LMR	0.974	0.920	1.032	0.374
PLR	1.004	1.001	1.008	0.010
SII	1.001	1.000	1.001	0.004
TyG	1.306	1.045	1.632	0.019

In the subsequent multivariate logistic regression model, each predictor demonstrated a significant and substantial association with DR risk: the odds ratio was 1.773 (95% CI: 1.561–2.014) for diabetes duration, 1.344 (95% CI: 1.196–1.511) for HbA1c, and 3.002 (95% CI: 2.087–4.319) for high BMI.

### 3.3 Nomogram construction and validation

**Figure 3:**
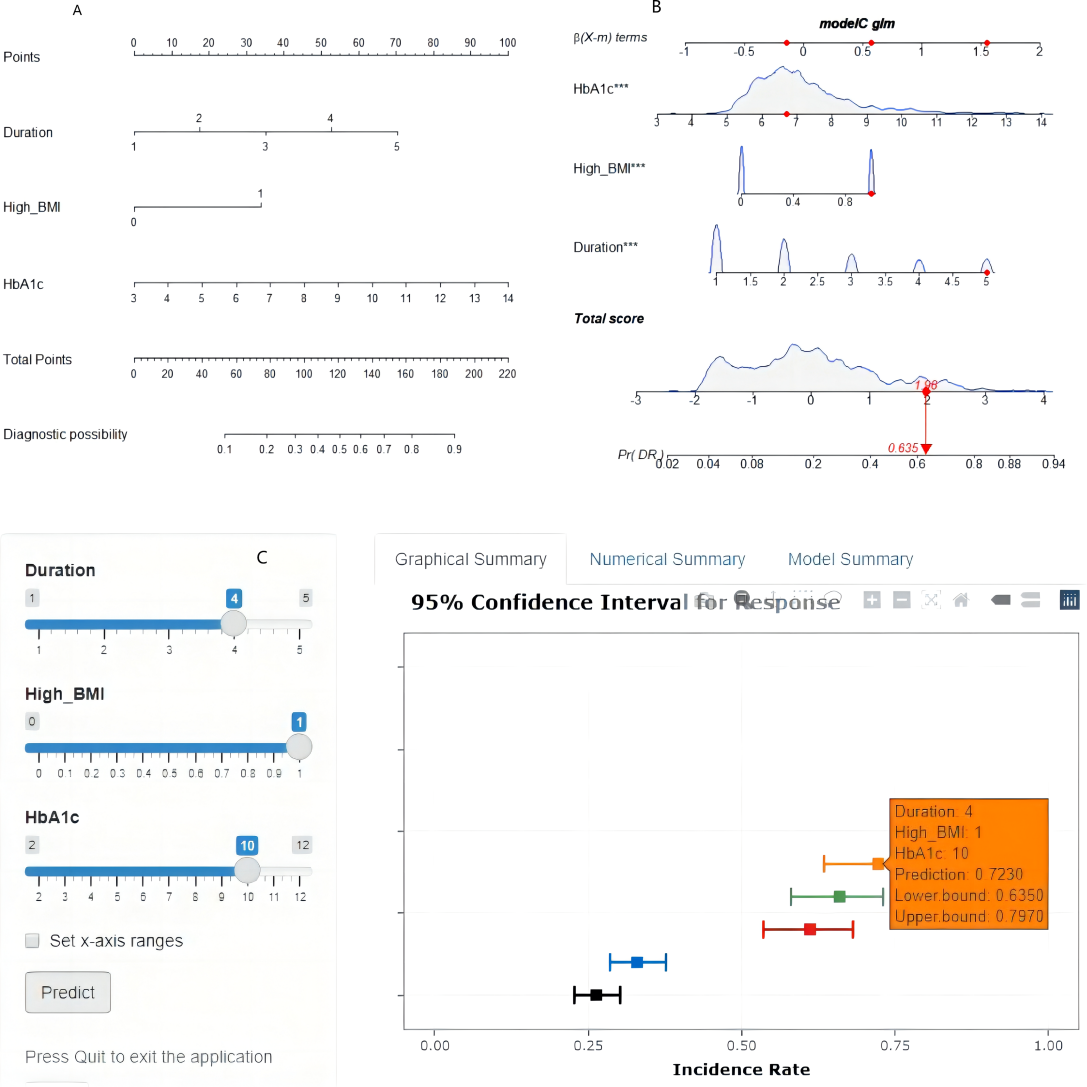
Nomogram and web-based implementation. **(A)** Printed nomogram for DR risk estimation. To use the nomogram, locate the patient’s diabetes duration category on the corresponding axis and draw a vertical line upward to the ‘Points’ bar to obtain the points for that variable. Repeat this for HbA1c and BMI status. Sum the points from all three variables, locate the total on the ‘Total Points’ axis, and draw a vertical line downward to obtain the predicted probability of DR. **(B)** Example case: a patient with high BMI (35 points), diabetes duration of 15–20 years (55 points), and HbA1c of 10% (65 points) has a total of 155 points, corresponding to a predicted DR probability of 72.3%. **(C)** Screenshot of the interactive online nomogram.

Based on the final logistic regression model, we constructed a visually interpretable nomogram to facilitate individualized DR risk assessment (**Figure 3A**). Detailed instructions for using the nomogram are provided in the **Figure 3** legend. This tool allows clinicians to quickly estimate a patient’s probability of DR by summing points assigned for their diabetes duration (categorized into 5-year increments: 0–5, 5–10, 10–15, 15–20, and >20 years), HbA1c level, and BMI status. The clinical applicability of the nomogram was further enhanced by the development of an interactive web application. Currently, the tool is deployed on a local institutional server to ensure data security and compliance with hospital policies. It is available upon reasonable request from the corresponding author for research or clinical use; interested users can contact Dr. Zhou Hairong (54574963@qq.com) to obtain access credentials. We are actively working toward making the tool publicly accessible via a dedicated website in the near future. Additionally, we are collaborating with the Shenzhen Longhua District People’s Hospital Group to pilot its integration into their electronic health record system, enabling automated risk calculation and alert generation for high-risk patients during routine consultations. The predictive accuracy of these tools was demonstrated through a representative case: for a patient with high BMI, 15–20 years of diabetes duration, and an HbA1c of 10%, both the nomogram and the web application yielded a consistent DR probability of 72.3% (**Figures 3B, C**).

### 3.4 Model performance evaluation

**Figure 4:**
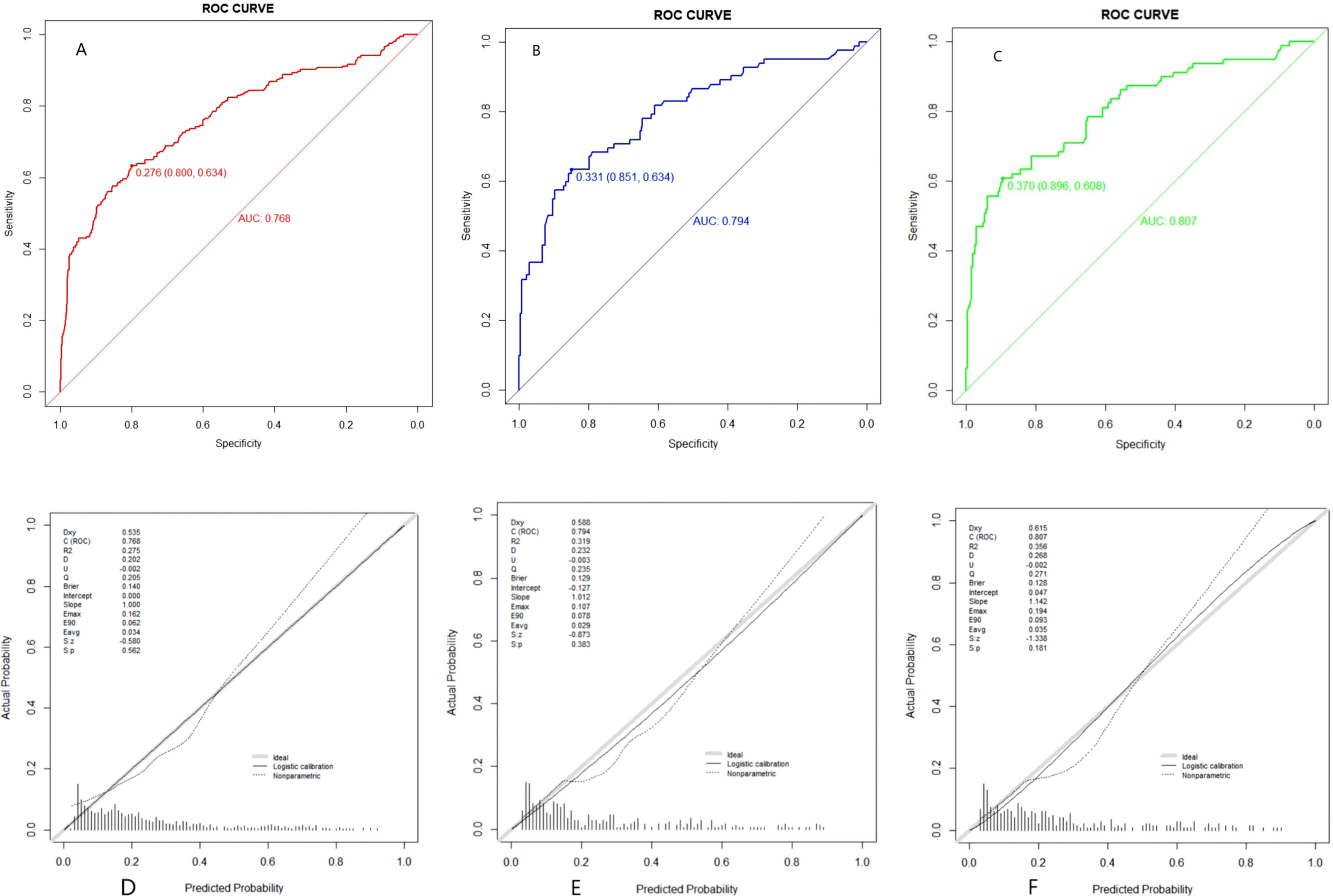
Receiver operating characteristic (ROC) curves and calibration plots. **(A–C)** ROC curves for training, internal validation, and external validation sets. **(D–F)** Corresponding calibration plots; dashed diagonal line indicates perfect calibration.

The model demonstrated consistent discriminatory ability across all datasets, with AUCs greater than 0.75 (all P < 0.001). Specifically, the AUC was 0.77 (95% CI: 0.73–0.81) in the training set, 0.79 (95% CI: 0.73–0.85) in the internal validation set, and 0.81 (95% CI: 0.75–0.87) in the external validation set (**Figures 4A–C**). The corresponding sensitivity and specificity, with their 95% CIs, were 0.63 (0.62–0.70) and 0.80 (0.79–0.87) in the training set, 0.63 (0.62–0.73) and 0.85 (0.84–0.92) in the internal validation set, and 0.61 (0.59–0.71) and 0.90 (0.88–0.96) in the external validation set, respectively. Calibration was adequate across all cohorts, as indicated by Brier scores below 0.15 and non-significant Hosmer-Lemeshow tests (all P > 0.05). The calibration plots (**Figures 4D–F**) display three curves: the ideal diagonal line, the logistic calibration curve, and the nonparametric calibration curve. In the training set, the ideal line and logistic calibration curve closely overlapped across the entire risk spectrum, indicating excellent model fit. Similarly, in both the internal and external validation sets, the logistic calibration curve remained closely aligned with the ideal line, with minimal deviation. The nonparametric curves also showed good agreement with both the ideal and logistic calibration lines across all three datasets. These findings confirm that the model produces well-calibrated risk estimates suitable for clinical decision-making.

**Figure 5:**
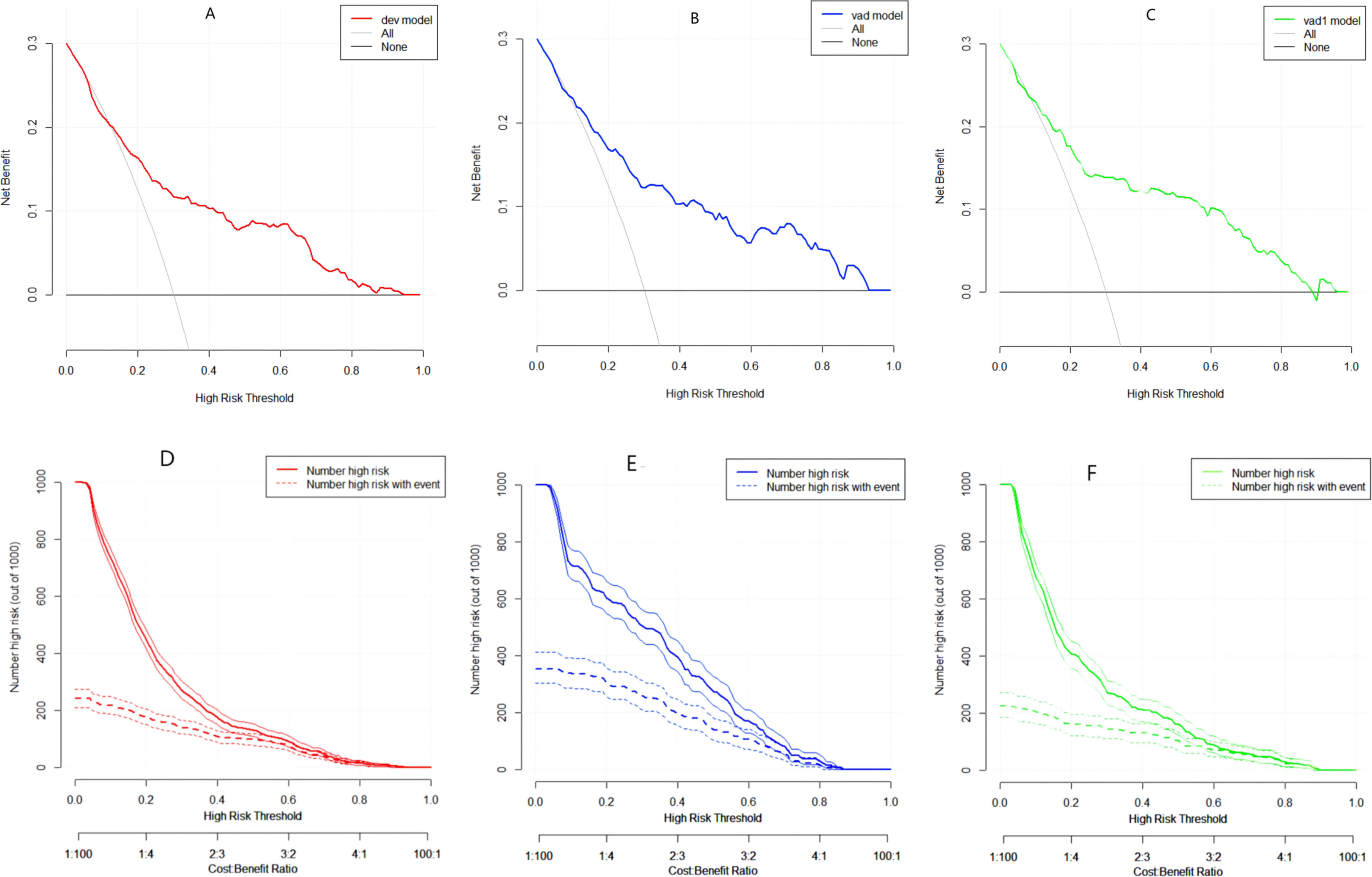
Decision curve analysis (DCA) and clinical impact curves (CIC). **(A–C)** DCA for the three datasets; y-axis represents net benefit. **(D–F)** CIC showing number of patients classified as high risk (red) and number of true DR cases (blue) across risk thresholds.

Decision curve analysis (**Figures 5A–C**) showed that the model provided a higher net benefit compared to “treat-all” or “treat-none” strategies across risk thresholds of 10% to 95%. Clinical impact curves (**Figures 5D–F**) revealed a 1:1 ratio of predicted versus actual DR cases when the threshold probability was set at 40% or higher.

## 4 Discussion

In this study, we developed and externally validated a pragmatic, web-based nomogram for diabetic retinopathy (DR) risk stratification tailored to primary care settings using an independent cohort from Nanjing. Using LASSO−penalized logistic regression, three readily available predictors—diabetes duration, HbA1c, and high body mass index (BMI ≥24 kg/m², Chinese criterion)—were selected from 41 candidate variables. The model demonstrated consistent discriminative performance across training (AUC 0.77, 95% CI: 0.73–0.81), internal validation (AUC 0.79, 95% CI: 0.73–0.85), and external validation (AUC 0.81, 95% CI: 0.75–0.87) cohorts. The overlapping confidence intervals indicate stable generalizability, and the reliance on universally accessible clinical parameters underscores its unique utility as a first−line DR screening tool in resource−limited settings. The modest incremental increase in AUC across cohorts likely reflects differences in case mix and data characteristics rather than systematic bias.

Compared with previously reported models, our approach offers superior feasibility without compromising predictive accuracy. At the optimal cutoff (0.370), the model achieved a sensitivity of 61% (95% CI: 55–67%) and a specificity of 90% (95% CI: 87–93%)—favorably comparable to the seven−parameter nomogram by Mo et al. (AUC 0.715) (24) and fundus photography−based deep learning classifiers (AUC = 0.71) (14). By requiring only three routine clinical variables, our model eliminates dependence on specialized equipment or expert graders, thereby addressing a critical implementation barrier in primary care (17, 18).

Decision curve analysis confirmed that the model provides net benefit exceeding both “refer−all” and “refer−none” strategies across threshold probabilities of 10–95%, with pronounced advantage in the intermediate−to−high risk range (30–70%). Clinical impact curves further revealed that at a risk threshold ≥40%, the ratio of predicted to observed DR cases approached unity. On this basis, we propose a tiered intervention framework: expedited ophthalmology referral for high-risk patients (≥40% predicted probability), intensified metabolic monitoring and annual fundus surveillance for intermediate-risk patients (20–40%), and routine annual evaluation for low-risk patients (<20%). The proposed risk thresholds were informed by the clinical impact curves (CIC, **Figures 5D–F**), which demonstrated that at a predicted probability threshold of ≥40%, the ratio of predicted high-risk patients to observed DR cases approached unity (1:1), supporting its use as a referral cutpoint. The 20–40% range was selected to identify intermediate-risk patients who may benefit from intensified monitoring without immediate referral, balancing clinical benefit against potential overburdening of specialty services. We acknowledge that these thresholds are exploratory and derived from retrospective data; they require prospective validation and formal cost-effectiveness analysis before widespread implementation. Future studies should assess whether these cutoffs optimize resource allocation and improve patient outcomes in real-world primary care settings.

The observed DR prevalence in our training cohort (24.1%) closely aligns with the 25% pooled estimate for Asian T2DM populations (25), and the 27% prevalence in the external validation cohort is consistent with contemporary community surveys (7). Nonetheless, geographic heterogeneity persists: Australia’s national screening program reported a 29% prevalence (26), whereas a 2023 tertiary hospital study in Southwest China documented only 21.7% among patients with comparable diabetes duration (19). These disparities likely reflect differences in healthcare access (community−based screening versus specialist−referred cohorts) and regional variations in glycemic control and adiposity profiles. Notably, even within Shenzhen’s highly regarded Community Health Center network—a flagship model for China’s primary care reform—the DR burden remains substantial. This paradox reinforces the imperative to transition from universal service provision toward precision risk stratification, even in economically advanced urban settings.

Diabetes duration exhibited a nonlinear, dose−dependent association with DR (OR 1.773, 95% CI: 1.561–2.014). Prevalence remained relatively stable between patients with <5 years (13.1%) and 5–10 years (14.3%) of disease (Δ +1.2%, P = 0.32), but increased sharply to 25.6% (Δ +11.3%, P < 0.001) in the 10–15 year stratum and reached 67% among those with >20 years of diabetes—a 4.1−fold elevation relative to patients with <10 years disease duration. This trajectory corroborates findings from the Saudi National Diabetes Registry (HR 2.34 for duration >10 years) (27) and multinational cohort data documenting a prevalence rise from 6.6% (0–5 years) to 63.0% (≥30 years) (28). Accordingly, we recommend semi−annual fundoscopic screening for T2DM patients with ≥10 years of disease duration to enable early detection of microvascular abnormalities and mitigate progression to proliferative DR (PDR) (8).

Glycated hemoglobin (HbA1c) emerged as a potent, independent risk factor; each 1% (11 mmol/mol) increment conferred a 34.4% increase in DR odds (OR 1.344, 95% CI: 1.196–1.511), consistent with prior Chinese and international studies (29, 30). The underlying pathophysiology involves three interrelated cascades (31): (i) metabolic derangement—chronic hyperglycemia induces oxidative stress and impairs endothelial mitochondrial function; (ii) inflammatory mediation—upregulated IL−6/TNF−α secretion compromises blood−retinal barrier integrity; and (iii) microvascular remodeling—PKC−β/NF−κB signaling drives capillary basement membrane thickening and retinal ischemia. Longitudinal evidence substantiates these mechanisms: patients with HbA1c >7% face a quadrupled DR risk compared to those at target (32–35), and the UK Prospective Diabetes Study (UKPDS) demonstrated that intensive glycemic control reduces DR incidence by 25% and vision−threatening events by 50% (36). We therefore advocate quarterly HbA1c monitoring with individualized targets (typically <7%), coupled with management of glycemic variability (amplitude ≤1.5 mmol/L) to minimize oxidative injury.

The association between BMI and DR remains controversial. A Shanghai−based cross−study reported a U−shaped relationship, with the lowest DR incidence observed at BMI 28–29.9 kg/m² (37). Research in Malaysian and Indian populations similarly suggested that while overweight and obesity increase diabetes risk, they paradoxically reduce DR occurrence (OR 0.82, 95% CI: 0.71–0.95) (38). A meta−analysis of 27 studies (total n >50,000) found no significant association when BMI was analyzed categorically (overweight: OR 0.89, 0.75–1.07; obesity: OR 0.97, 0.73–1.30) or continuously (OR per 1 kg/m²: 0.99, 0.97–1.01) (39). Conversely, emerging data from India (2025) demonstrated significantly higher BMI in patients with proliferative DR (40), and our study identified high BMI (≥24 kg/m²) as a strong independent risk factor (OR 3.002, 95% CI: 2.087–4.319), corroborating NHANES−based findings (41).

Several factors may explain these discrepancies: ethnic differences in visceral adiposity distribution, heterogeneous BMI categorization (WHO versus Asian criteria), and inconsistent confounding control—fewer than 40% of reviewed studies adjusted for diabetes duration and HbA1c (39). Mechanistically, high BMI may promote retinopathy through compensatory hyperinsulinemia−mediated endothelial injury (42), chronic inflammation driven by adipokine imbalance (e.g., elevated TNF−α/IL−6) (43), oxidative stress via polyol pathway activation and advanced glycation end−product accumulation (44), and gut microbiota dysbiosis affecting the gut−retina axis (45). Future research should adopt standardized Asian BMI cutoffs, incorporate central obesity measures (waist circumference or waist−to−hip ratio), and apply flexible modeling techniques (e.g., restricted cubic splines) to delineate dose−response relationships. Clinically, our findings caution against the “obesity paradox” in DR and underscore that weight management remains a priority even in patients with apparently adequate glycemic control.

This study has several methodological and translational strengths. First, the model was developed using real−world EHR data aggregated from 45 community health centers and validated in an independent, cross−provincial cohort, substantiating its generalizability across diverse primary care populations in China (46). Second, the two−stage feature selection strategy—univariate screening followed by LASSO regression—mitigates overfitting and ensures model parsimony without compromising predictive stability. Third, the final model relies on only three routinely documented parameters, conferring immediate scalability at negligible incremental cost (47). Fourth, the interactive web−based nomogram enables real−time risk visualization at the point of care, facilitating shared decision−making and seamless integration into clinical workflows.

Several limitations merit acknowledgment. First, the retrospective design introduces potential selection bias and unmeasured confounding, despite rigorous adjustment for known covariates. Prospective multicenter validation using population−based sampling is warranted to confirm model performance and real−world impact. Second, both derivation and validation cohorts were drawn from urban primary care facilities in relatively developed regions of China; extrapolation to rural populations—where diabetes management infrastructure, health literacy, and referral pathways differ substantially—requires caution. Third, the moderate sample size precluded stage−specific analyses (e.g., non−proliferative versus proliferative DR). Larger cohorts are needed to develop progression−stage−specific models that can further refine referral prioritization. Fourth, BMI was dichotomized at the Chinese overweight threshold (≥24 kg/m²) to maximize clinical simplicity; however, this categorization may obscure nonlinear relationships and attenuate effect estimates. Future iterations of the model could incorporate continuous BMI or spline transformations to capture more nuanced risk gradients. Fifth, although our model performed well in two independent Chinese cohorts, its generalizability to other ethnic groups or healthcare systems remains to be tested. Future studies should focus on external validation in diverse populations. While our nomogram prioritizes simplicity and interpretability, we acknowledge that more complex machine learning algorithms—such as random forests or gradient boosting machines—could potentially improve predictive accuracy by capturing nonlinear effects and interactions. However, such models often function as ‘black boxes,’ which may hinder clinical adoption in primary care settings where transparency is essential. Moreover, the incremental benefit of these methods over our parsimonious model remains to be demonstrated in prospective comparisons. Future research should explore hybrid approaches that balance interpretability with predictive power and assess whether the added complexity translates into meaningful improvements in risk stratification and patient outcomes. Notwithstanding these limitations, the model’s immediate clinical utility remains intact, and the identified constraints delineate clear pathways for future translational optimization.

## 5 Conclusions

In summary, we have developed and multi-regionally validated a pragmatic, three-parameter online nomogram for DR risk stratification that relies exclusively on variables universally available in primary care EHRs. The model exhibits robust discrimination, excellent calibration, and consistent net clinical benefit across a wide spectrum of risk thresholds. By enabling low-cost, real-time identification of high-risk T2DM patients, this tool holds promise for reducing the burden of preventable blindness and optimizing the allocation of specialist resources within integrated healthcare systems.

## Data Availability

The original contributions presented in the study are included in the article/supplementary material. Further inquiries can be directed to the corresponding author.
